# *In Vitro* Antibacterial Activity of Ibuprofen and Acetaminophen

**DOI:** 10.4103/0974-777X.62880

**Published:** 2010

**Authors:** Ali Abdul Hussein S. AL-Janabi

**Affiliations:** *Department of Clinical Laboratory, University of Karbala, Collage of Pharmacy, Iraq*

**Keywords:** Acetaminophen, Antibacterial, Ibuprofen

## Abstract

**Background:**

Ibuprofen and acetaminophen are common chemical agents that have anti-inflammatory, antipyretic, and analgesic activity.

**Aims:**

To detect any potential antibacterial effects of ibuprofen and acetaminophen on pathogenic bacteria.

**Materials and methods:**

Ibuprofen and acetaminophen were tested for antibacterial activity against seven isolates of bacteria including gram positive bacteria (*Staphylococci aureus* and *Bacillus subtilis*) and gram negative bacteria (*E. coli, Enterobacter aerogenes, Enterobacter cloacae, Salmonella typhi and Paracoccus yeei*). Spectrophotometer assay was applied to determine the antibacterial activities of ibuprofen and acetaminophen. Three controls were included in this study: Ampicilline sodium (20 μg/ml); cefotaxime sodium (20 μg/ml) and chemical free medium.

**Results:**

*Staphylococcus aureus* and *Paracoccus yeei* were susceptible to lower concentrations of ibuprofen and acetaminophen (MIC=1.25 mg/ml), while two strains of *Enterobacter* exhibited resistance to these agents.

**Conclusions:**

Ibuprofen and acetaminophen showed a potential antibacterial effect on isolated strains of bacteria. They had the same ability to inhibit bacterial growth.

## INTRODUCTION

Acetaminophen is a synthetic non-opiate derivative of ϱ-aminophenol, while ibuprofen is one of phenyl propionic acid derivatives.[1] These chemical agents have anti-inflammatory, antipyretic, and analgesic activity in both animals and humans.[2–4] Available information about the relationship between bacteria and ibuprofen or acetaminophen is focused on the anti-inflammatory action of these agents on immune system that stimulated by bacterial infections and not on the antibacterial activities. Ibuprofens found to be limited the effect of *E. coli* endotoxin on several physiological activities of rabbits[5] and human.[6] It also reduces the inflammation in mouse lung resulting from *Pseudomonas aerogenosa* infection with no effect on the bacteria itself.[7] Otherwise, ibuprofen showed no antibacterial effects on *Campylobacter pylori* in human body[8] and on *Mycobacterium tuberculosis* in mice.[9] Complexes of acetaminophen with Co, Ni or Fe element (not acetaminophen alone) revealed variable inhibitory effects on *E. coli*, while *Serratia* and *Bacillus subtilis* didn't affect by any of these complexes.[10] Thus, direct action of ibuprofen and acetaminophen on bacterial cells did not clearly illustrate until now.

Although few studies found that ibuprofen and acetaminophen had significant effects to reduce some of body disorders after bacterial infections, antibacterial action of these agents are not clear for many species of pathogenic bacteria. Thus, the main goal of this study was to detect any additional activities of ibuprofen and acetaminophen rather than anti-inflammatory activity by testing their ability to inhibit many pathogenic bacteria.

## MATERIALS AND METHODS

### Strains

Seven strains of bacteria were recently clinical isolated from inpatients (30-38 years) at AL-Hussein general Hospital of Karbala province. The ethical approval was obtained from the University of Karbala (College of Pharmacy). Throat swabs and stool samples were cultured on Mueller-Hinton agar (HiMedia, Mumbai- India) and incubated at 35° C for 24 h. Diagnosis was performed using API 20 system (Biomérieux, Netherlands-France) in addition to gram staining and morphological criteria.

The isolated strains were *: Staphylococci aureus, E. coli, Bacillus subtilis, Enterobacter aerogenes, Enterobacter cloacae, Salmonella typhi and Paracoccus yeei.*

### Chemical agents

Ibuprofen and acetaminophen were supplied from Arabia industry drugs (AID, Baghdad-Iraq). Ampicilline sodium and cefotaxime sodium were supplied by KonTam pharmaceuticals co. Zhongshan-China.

### Antibacterial assays

Standard culture of bacteria for antibacterial assay was prepared in Mueller-Hinton broth (HiMedia, Mumbai-India) equivalent to a 0.5 MacFarland Nephelometer standard (reading to 1×10^8^ cfu/ml) and diluted 1:10.

For obtaining stock solutions, ibuprofen and acetaminophen were dissolved in methanol. Ampicilline sodium and cefotaxime sodium were dissolved in sterile distilled water. Drug concentrations were serial two-fold dilutions ranging from 5 to 0.312 mg/ml.

Spectrophotometer assay was applied to determine the antibacterial activities of ibuprofen and acetaminophen. Tubes of Mueller-Hinton broth containing various concentrations of ibuprofen and acetaminophen were prepared. These tubes were inoculated with standard culture of each strain (50 μl to each milliliter of broth). All tubes were incubated at 35 °C for 24 h. Optical density of bacterial growth was measured by spectrophotometer (Optima-SP-300, Kyoto-Japan) at wavelength of 450 nm.[11]

Three controls were included in this study: Ampicilline sodium (20 μg/ml); cefotaxime sodium (20 μg/ml) and chemical free medium. Each experiment was repeated three times with triplicates of each concentration for statistical analysis.

### Determination of minimum inhibitory concentration

Minimum inhibitory concentrations (MICs) were determined as described by NCCLS.[12] Chemical agents were twofold diluted in Mueller-Hinton broth. A 100 μl of each dilution was dispensed in well of microdilution plates (96-wells). Well was inoculated with 50 μl of standard culture medium. The inoculated plates were incubated at 35 ° C for 24 h and examined for visible growth in order to determine MIC. Three previous controls were also included.

### Statistical analysis

Data were statistically analyzed by using two-way variance of analysis (ANOVA) with less significant difference (L.S.D.) at *P*<0.05.

## RESULTS

In addition to pharmacological applications of ibuprofen and acetaminophen as anti-inflammation and antipyretic drugs, investigation for other functions mainly against bacteria was performed.

**Figure 1:**
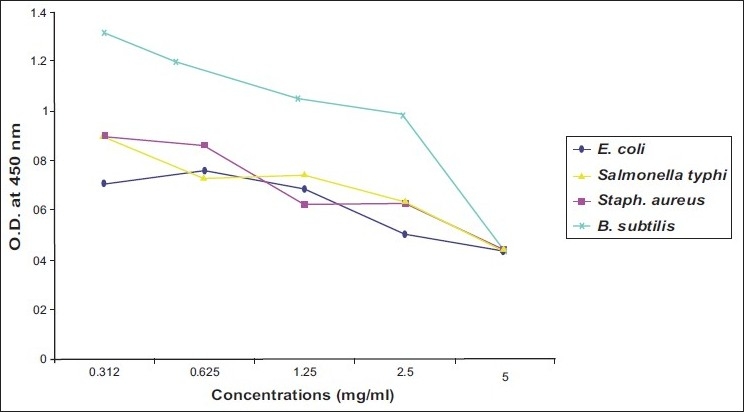
Effect of different concentrations of ibuprofen on *E. coli, Salmonella typhi, Staph aureus* and *B. subtilis*

**Figure 2:**
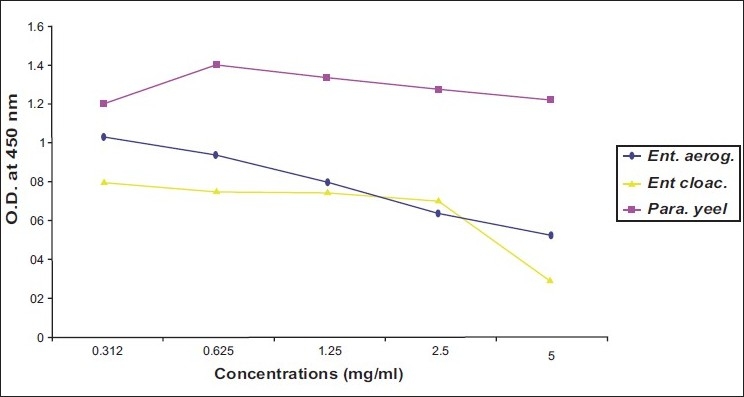
Effect of ibuprofen on *Enterobacter aerogenes, Enterobacter cloacae* and *Paracoccus yeei*

**Figure 3:**
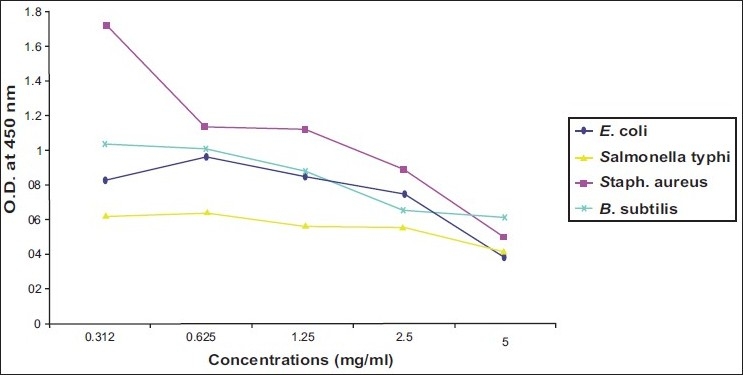
Effect of different concentrations of Acetaminophen on *E. coli, Salmonella typhi, Staph aureus* and *B. subtilis*

**Figure 4:**
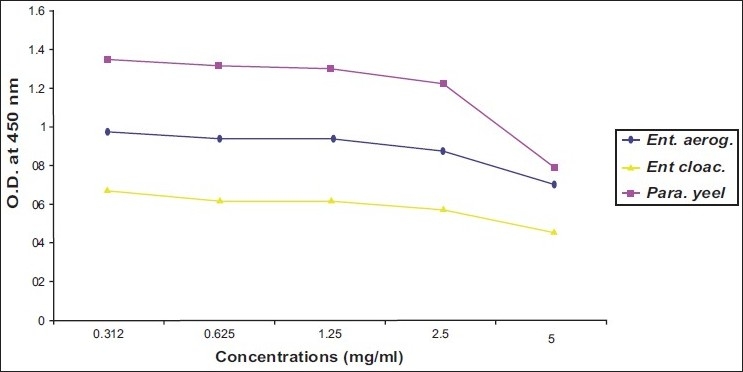
Effect of different concentrations of Acetaminophen on *Enterobacter aerogenes, Enterobacter cloacae* and *Paracoccus yeei*

Activity of ibuprofen and acetaminophen on isolated bacteria was proximally progressed in parallel direction. Two of gram negative bacteria (*E. coli* and *Salmonella typhi*) and one of gram positive bacteria (*B. subtilis*) revealed susceptibility to both tested agents at MIC of 2.5 mg/ml [Figure 1 and 3]. While two strains of *Enterobacter* needed more concentrations of ibuprofen and acetaminophen (MIC=5 mg/ml) to inhibit [Table 1] [Figure 2 and 4].

**Table 1:** MICs of ibuprofen and acetaminophen in isolated bacteria

Strains	Amp. (μg/ml)	Cefot. (μg/ml)	Ibuprofen concentrations (mg/ml)					Acetaminophen concentrations (mg/ml)				
				
	20	20	5	2.5	1.25	0.625	0.312	5	2.5	1.25	0.625	0.312
*E. coli*	−	−	−	−	+	+	+	−	−	+	+	+
*Sal. typhi*	−	−	−	−	+	+	+	−	−	+	+	+
*Ent. cloacae*	−	−	−	+	+	+	+	−	+	+	+	+
*Ent. aerog.*	−	−	−	+	+	+	+	−	+	+	+	+
*P. yeei*	−	−	−	−	−	+	+	−	−	−	+	+
*Staph. aureus*	−	−	−	−	−	+	+	−	−	−	+	+
*B. subtilis*	−	−	−	−	+	+	+	−	−	+	+	+

+ clear growth

− no growth

As illustrated in four figures, *Paracoccus yeei* and *S. aureus* showed more susceptibility to ibuprofen and acetaminophen than other isolates at MIC of 1.25 mg/ml with significant differences (*P*<0.05) from control (free media). However, ibuprofen and acetaminophen had less effective on all of isolated bacteria compared with ampicilline and cefotaxiam [Table 1].

## DISCUSSION

Among non-steroidal anti-inflammatory drugs (NSAID), ibuprofen and acetaminophen are the most popular drugs. These medications are of the common self-administered drugs to date.[13] Although production from difference sources, similarity in action between ibuprofen and acetaminophen on many isolated strains of bacteria may related to the same side of action in bacterial cells. The site of action of ibuprofen and acetaminophen in human body is determined by inhibiting the synthesis of prostaglandin through effect on cyclooxygenase enzyme.[1,6] This mechanism is not available in microorganisms due to lack the presence of cyclooxygenase. Otherwise, killing activity of ibuprofen in *Candida* cells was found through direct damage in their cytoplasmic membrane.[14]

Among seven strains of isolated bacteria, *Enterobacter aerogenes* and *Enterobacter cloacae* exhibited more resistance to ibuprofen and acetaminophen. These strains are the species most commonly encountered in clinical specimens. They are part of commensal enteric flora.[15] *Enterobacter cloacae* found to be resistance to many antibiotics. It needs high MIC of pexiganan to inhibit[16] with the ability to survive for long periods after postantibiotic effect of meropenem.[17] Also the resistant to cephalosporins had been observed due to the production of a stably cephalosporinase.[18] This enzyme is related to β-lactamase group that expressed by plasmid.[19] It considers the most important mechanism of resistance in *Enterobacter* strains.[20]

*Paracoccus yeei* is a gram negative rod bacterium that could be isolated from various human wound infections and blood.[21]. It exhibited with *S. aureus* more susceptibility to ibuprofen and acetaminophen. According to our results, susceptibility of bacterial strains to ibuprofen and acetaminophen may encourage usage of these chemical compounds as assistant agents with other standard antibacterial drugs. Thus, the information about disadvantage effects of ibuprofen or acetaminophen on the therapeutical activities of some antibiotic when administrated as antipyretic agents may change.[22] However, higher peak level of ceftizoxime was detected in plasma above the minimum therapeutic concentrations for along duration after co-administrated of ceftizoxime along with acetaminophen.[23] Moreover, ibuprofen enhanced the activity of pyrazinamide during the initial phase of tuberculosis treatment in mouse model.[9]

## CONCLUSION

Ibuprofen and acetaminophen showed the same ability to inhibit the growth of bacteria. They had a potential antibacterial effect on isolated strains of bacteria. This *in vitro* activity needs to develop for *in vivo* test and further studies are demanding to confirm our results.

## References

[1] McEvoy GK, Litvak K, Welsh OH, Dewey DR, Fong PA, Douglas PM (1993). AHFS drug information. American society of hospital pharmacists.

[2] Mainous AG, Pearson WS (2003). Aspirin and ibuprofen: Potential mediators of the cardiovascular risk due to smoking. Fam Med.

[3] Polidori G, Titti G, Pieragostini P, Comito A, Scaricabarozzi I (1993). A comparison of nimesulide and paracetamol in the treatment of fever due to inflammatory disease of the upper respiratory tract in children. Drugs.

[4] Walsh A (2006). Childhood fever.

[5] Celik I, Akbulut A, Kilic SS, Rahman A, Vural P, Canbaz M, Felek S (2002). Effects of ibuprofen on the physiology and outcome of rabbit endotoxic shock. BMC infectious diseases.

[6] Bernard GR, Wheeler AP, Russell JA, Schein R, Summer WR, Steinberg KP (1997). The effects of ibuprofen on the physiology and survival of patients with sepsis. New Engl J Med.

[7] Sordelli DO, Cerquetti MC, el-Tawil G, Ramwell PW, Hooke AM, Bellanti JA (1985). Ibuprofen modifies the inflammatory response of the murine lung to Pseudomonas aerogenosa. Eur J Respire Dis.

[8] Graham DY, Klein PD, Opekun AR, Smith KE, Polasani RR, Evans DJ (1989). In vivo susceptibility of *Campylobacter pylori*. Am J Gastroenterol.

[9] Byrne ST, Denkin SM, Zhang Y (2007). Aspirin and ibuprofen enhance pyrazinamide treatment of murine tuberculosis. J Antimicrob Chemoth.

[10] Lawal A, Obaleye JA (2007). Synthesis, characterization and antibacterial activity of aspirin and paracetamol-metal complexes. Biokem.

[11] Domínguez MC, de la Rosa M, Borobio MV (2001). Application of a spectrophotometric method for the determination of post-antibiotic effect and comparison with viable counts in agar. J Antimicrob Chemoth.

[12] NCCLS (2003). Methods for dilution antimicrobial susceptibility testing for bacteria that grow aerobically; approved standard-sixth edition. NCCLS document M7-A6.

[13] Johnson JM (2008). Over-the-counter overdoses: A review of ibuprofen, acetaminophen, and aspirin toxicity in adults. Advan Emerg Nur J.

[14] Pina-Vaz C, Sansonetty F, Rodrigues AG, Martinez-De-Oliveira J, Fonseca AF, Mardh P (2000). Antifungal activity of ibuprofen alone and in combination with fluconazole against Candida species. J Med Microb.

[15] Winn W, Allen S, Janda W, Koneman E, Procop G, Schreckenberger P, Woods G (2006). Koneman's color atlas and textbook of diagnostic microbiology.

[16] Ge Y, MacDonald, Holroyd KJ, Thornsberry C, Wexler H, Zasloff M (1999). *In vitro* antibacterial properties of pexiganan, an analog of magainin. J Antimicrob Chemoth.

[17] Mackenzie FM, Gould IM, Chapman DG, Jason D (1994). Postantibiotic effect of meropenem on members of the family Enterobacteriaceae determined by five methods. J Antimicrob Chemoth.

[18] Stearne LE, van Boxtel D, Lemmens N, Goessens WH, Mouton JW, Gyssens IC (2004). Comparative study of the effects of ceftizoxime, piperacillin, and piperacillin-tazobactam concentrations on antibacterial activity and selection of antibiotic-resistant mutants of Enterobacter cloacae and Bacteroides fragilis *in vitro* and in vivo in mixed-infection abscesses. J Antimicrob Chemoth.

[19] Ikonomidis A, Spanakis N, Poulou A, Pournaras S, Markou F, Tsakris A (2007). Emergence of carbapenem-resistant Enterobacter cloacae carrying VIM-4 metallo-β-lactamase and SHV-2a extended-spectrum β-lactamase in a conjugative plasmid. Microb Drug resist.

[20] Ehrhardt AF, Sanders CC (1993). Beta-lactam resistance amongst Enterobacter species. J Antimicrob Chemoth.

[21] Funke G, Frodl R, Sommer H (2004). First comprehensively documented case of Paracoccus yeei infection in a human. J Clin Microb.

[22] Mandell GL, Coleman EJ (2002). Effect of antipyretic agents on uptake, transport, and release of antimicrobial agents by human polymorphonuclear leukocytes. J Infect Dis.

[23] Singh R, Chaudhary RK, Dumka VK (2008). Influence of paracetamol on the pharmacokinetics and dosage regimen of ceftizoxime in cross bred calves. Isra J Veterin Med.

